# Editorial: Blood biomarkers in cardiovascular diseases

**DOI:** 10.3389/fcvm.2025.1587259

**Published:** 2025-04-09

**Authors:** Yan Xu, Qiang Liu, Wen-Jun Tu

**Affiliations:** ^1^ Department of Neurosurgery, Beijing Tiantan Hospital, Capital Medical University, Beijing, China; ^2^ Institute of Radiation Medicine, Chinese Academy of Medical Sciences & Peking Union Medical College, Tianjin, China; ^3^ Translational Laboratory of Clinical Medicine, Chinese Institute for Brain Research & Beijing Tianan Hospital, Beijing, China

**Keywords:** cardiovascular diseases, blood bimarkers, editor, inflammation, metabolic dysregulation

**Editorial on the Research Topic**
Blood biomarkers in cardiovascular diseases

Cardiovascular diseases (CVD) remain the leading cause of morbidity and mortality worldwide, underscoring the urgent need for improved diagnostic, prognostic, and therapeutic strategies (1). Blood biomarkers have emerged as indispensable tools in this regard, offering insight into the pathophysiological mechanisms underlying CVD and providing clinically relevant information for risk stratification and treatment optimization (2). These biomarkers not only aid in the monitoring and analysis of the spectrum but also enhance our understanding of the underlying pathogenic mechanisms of CVD (3, 4). This special issue explores recent advancements in blood biomarkers for CVD, featuring seven studies that extend our understanding beyond mere associations and move toward mechanistic insights, clinical applicability, and predictive modeling (https://www.frontiersin.org/research-topics/57019/blood-biomarkers-in-cardiovascular-diseases). The selection of these studies is driven by their relevance to contemporary cardiovascular research, their methodological rigor, and their potential to impact clinical practice.

One of the highlighted biomarkers in this issue is red cell distribution width (RDW), a measure of erythrocyte volume heterogeneity traditionally used in the classification of anemia (3). While RDW has been associated with adverse cardiovascular outcomes, the causal relationship remains uncertain (4). Pan et al. conducted a large population-based cohort study, integrating individual follow-up data and genetic analyses, to assess the prognostic and etiological roles of RDW in cardiovascular and metabolic diseases. Their findings confirmed strong associations between RDW and stroke, atrial fibrillation, heart failure, venous thromboembolism, diabetes, chronic kidney disease (CKD), and all-cause mortality. However, the absence of a significant association between genetically determined RDW and cardiovascular events suggests that RDW may act as a marker of systemic dysfunction rather than a direct causal factor. Notably, their observation of a negative correlation between RDW and diabetes, both phenotypically and in polygenic risk scores, challenges prior assumptions and calls for further mechanistic studies to elucidate the underlying biological pathways.

Inflammation is a central driver of cardiovascular pathology, influencing the progression of atherosclerosis, heart failure, and thrombosis (5). Several systemic inflammatory markers have been proposed as potential predictors of adverse cardiovascular events. In a large-scale cross-sectional study, Huang et al. assessed the predictive value of five inflammatory indices: the neutrophil-to-lymphocyte ratio (NLR), platelet-to-lymphocyte ratio (PLR), systemic immune-inflammation index (SII), systemic inflammatory response index (SIRI), and aggregate inflammatory systemic index (AISI). Their results demonstrated robust correlations between these markers and heart failure incidence, highlighting their potential utility in risk stratification. However, as these markers are nonspecific and influenced by comorbid conditions, further longitudinal studies are needed to validate their predictive value and establish their role in guiding anti-inflammatory interventions.

Myocardial ischemia and infarction remain key contributors to CVD burden, particularly in regions where risk factors such as diabetes and hypertension are prevalent (6). In this context, Luo et al. explored the clinical significance of leucine-rich α-2 glycoprotein 1 (LRG1), a hepatocyte-derived secretory glycoprotein, in ST-segment elevation myocardial infarction (STEMI). Their study revealed dynamic changes in plasma LRG1 levels from admission to post-event recovery, with peak levels correlating with Th17 cell activity and major adverse cardiovascular events (MACE) risk. These findings align with emerging evidence implicating LRG1 in vascular inflammation and remodeling, positioning it as a potential biomarker for risk stratification and therapeutic targeting in acute coronary syndromes. However, further interventional studies are required to determine whether modulating LRG1 pathways can improve clinical outcomes.

Beyond ischemic events, metabolic dysregulation plays a pivotal role in cardiovascular risk. Yin et al. investigated the impact of admission glucose (AG) levels on MACE incidence within 30 days of acute cardiac chest pain presentation. Their findings reaffirm the prognostic significance of hyperglycemia in acute cardiovascular settings, emphasizing the need for early glucose monitoring and management. While previous studies have linked stress hyperglycemia to poor cardiovascular outcomes, the underlying mechanisms remain incompletely understood. This study underscores the importance of personalized glycemic control strategies, particularly in high-risk individuals.

The intersection of infectious disease and cardiovascular pathology has gained attention following the COVID-19 pandemic, which has been associated with increased thrombotic complications (4). Benatti et al. identified four clinical variables associated with heightened cardiovascular risk following SARS-CoV-2 infection: prior thrombotic or cardiovascular events, a BRIXIA score ≥3, an NLR ≥3.3, and an estimated glomerular filtration rate (eGFR) <45 ml/min/1.73 m^2^. Their findings reinforce the need for post-infection cardiovascular monitoring, particularly in individuals with preexisting risk factors. However, the mechanistic links between viral infections and cardiovascular complications remain an area of ongoing investigation, necessitating further studies to refine risk prediction models.

Two additional studies in this issue focus on physiological and socioeconomic determinants of CVD. Chen et al. examined the trajectory of blood pressure changes in an elderly Chinese cohort, demonstrating that longitudinal increases in systolic and diastolic blood pressure correlate with heightened CVD risk. Their findings highlight the need for dynamic risk assessment models that incorporate blood pressure trends rather than static measurements. Meanwhile, Zhou et al. analyzed data from the National Health and Nutrition Examination Survey (NHANES) (2007–2018) to explore the relationship between eGFR and CVD risk. Their study revealed a significant nonlinear association, with a threshold effect at an eGFR of 9.93. Below this threshold, each 10-unit increase in eGFR was associated with a 13% reduction in CVD risk, whereas above this threshold, no significant correlation was observed. These findings underscore the complex interplay between renal function, anemia status, and socioeconomic factors in cardiovascular risk stratification.

These studies collectively highlight the dynamic evolution of blood biomarkers in cardiovascular research. Although biomarkers possess significant potential for diagnosis and prognosis, their successful clinical implementation demands careful attention to factors such as individual variability, assay standardization, and seamless integration with existing risk assessment models (7). Moving forward, research efforts should focus on validating biomarkers across diverse populations, delving into the mechanistic underpinnings of biomarker pathways, and conducting interventional trials to evaluate their therapeutic impact. As the field progresses toward precision medicine, the convergence of biomarker discovery with cutting-edge technologies—such as multi-omics profiling and artificial intelligence—offers immense potential to revolutionize cardiovascular care. By honing biomarker-driven approaches, we can improve early detection, tailor treatments to individual needs, and ultimately elevate patient outcomes in the battle against cardiovascular disease (CVD).
